# Diseases of Tropical Climates

**Published:** 1898-07

**Authors:** 


### DISEASES OF TROPICAL CLIMATES.

Just at this time our attention is called to the diseases prevalent in tropical climates which are likely to attack our army, perhaps proving more formidable foes than the fiery Spaniards. Dr. T. S. Dabney read a paper on this subject recently before the New Orleans Parish Medical Society (*New York Medical Journal*, June 18), which is of interest and value to Northern physicians who have had little or no practical and personal knowledge of the diseases met with in southern countries.

Contrary to what one might expect, he says that heat-stroke is not peculiar to the tropics and we have little to fear from it if proper attention is paid to the predisposing causes. Anything that tends to depress the vital powers is largely responsible for heat-stroke, particularly during the rainy season when there is great humidity, continuous high temperature and lack of cooling breezes. Soldiers should not be required to do much hard work for at least a month after landing in a tropical country. The treatment of heat-stroke is practically the same everywhere, cold baths with vigorous rubbing with crash gloves. Iced enemata often proves valuable. The patient should be taken out of the bath when his temperature reaches 103° F.; he should be wrapped in a blanket and hot bottles placed around him and a stiff drink of whisky generally be given.

Tropical anemia is due to an intestinal parasite, a reddish brown worm from eight to ten millimetres in length. By strict sanitary measures they can be kept from the alimentary canals of our soldiers. Any treatment must consist in the elimination of the parasite and iron and bitter tonics for the secondary anemia afterwards. Male fern has been recommended but often fails. Thymol is used successfully in most cases. A brisk purge should always be given first and large saline enemata afterward in order to wash away the ova and larvæ in the rectum.

Smallpox flourishes everywhere in Cuba, and should the soldiers suffer from this the surgeons should be held responsible. Dr. Dabney thinks we can profit greatly from the German method of always making at least three insertions of the lymph in vaccinating, thereby increasing the chances of success. Vaccination, when perfectly successful, loses its effect in many persons in a few years and in some in a few months, so we should vaccinate the unvaccinated, revaccinate the vaccinated and re-revaccinate the revaccinated.

Yellow fever has been written on fully by various physicians, but the Sternberg bichloride treatment is especially recommended. This in connection with the daily saline enemata, abundance of cool water, fresh air and sunshine, and with strict attention to diet will give such good results as to rob the disease of its terror.

Beri-beri, both in the "wet" and "dry" varieties (paralytic and dropsical), is a very striking disease. Upon casual observation it might be mistaken for profound malarial toxemia, ankylostomiasis, alcoholic or arsenical poisoning. It is essentially an endemic multiple neuritis, characterized by great anemia, irregular, rapid, palpitating action of the heart, with marked dyspnea and cyanosis upon the slightest exertion. In the "wet" variety, edema, usually commencing on the dorsum of the foot, rapidly extends, filling the serous cavities, especially the pericardium. General anasarca soon supervenes. Various motor paralyses and atrophies occur, the wrist and ankle drop and atrophy of legs below the knee being most pronounced. At times the diaphragm itself is paralyzed. The ankle drop gives a peculiar gait that, once seen, can never be mistaken. Many authors still consider that certain articles of diet—notably rice and fish, especially tainted fish—serve as predisposing causes. The treatment demands first the relief of the burning, stinging and well-nigh unbearable pains. Drugs of the coal-tar series, cautiously used and in moderate doses, often afford marked relief. Opium may have to be resorted to. Constipation, a prominent symptom, will have to be met with proper cathartics, dropsies by the use of diuretics and diaphoretics. Strychnine, digitalis, camphor and stropanthus will be of utility in stimulating the heart, which should receive the most earnest attention. Galvanism in some instances affords excellent results. The prompt removal of the patient to the mountains, with fresh air, sunshine, wholesome food and fresh water, will prove the best treatment. Dr. Dabney thinks that our soldiers will not be likely to contract this disease unless they fall into Spanish prisons.

The malarias of the tropics may be divided into two great classes—the benign (calenturas) and the bad (the pernicious fevers). The first correspond to our ordinary tertian or quotidian type, are very mild or moderately severe, and always yield promptly to moderate doses of the sulphate of quinine administered per os, in solution or powder. If, however, these fevers are not treated promptly they are liable to change their type and become pernicious. The initial chill may be wanting but is liable to be severe, and the liver and spleen are usually enlarged. The chill usually occurs in the daytime, seldom earlier than 8 A. M. and not often after 4 P. M. The treatment should be vigorous, beginning with a good purge, mercurial preferred, and followed by seven grains and a half of sulphate of quinine every hour or two until decided cinchonism occurs; usually three doses will be sufficient. Five grains of acetanilid or phenacetin seem to enhance the value of the drug and add to the patient's comfort. Usually one dose of acetanilid is sufficient. The patient should be kept in bed, the bowels, skin and kidneys looked after, and moderate cinchonism kept up after the subsidence of the fever. On the third day seven grains and a half of quinine combined with five grains of Dover's powder or acetanilid should be given four hours before the chill time, and five grains of quinine every hour afterward, until twenty-five to thirty grains have been given. Fifteen grains daily in three doses should be administered for four or five days and then some bitter tonic, or iron and arsenic, for from ten days to two weeks. In many cases less heroic treatment will succeed, but unfortunately it is hard to tell at the beginning of the disease which these may be.

The pernicious form is due to infection from the aestivo-autumnal or crescentric parasites which invade all organs of the body. This is always accompanied by coma. When these parasites invade the gastro-intestinal canal we have intestinal malaria masquerading under the guise of a vicious dysentery or choleraic diarrhea. When the meninges and brain swarm with them, menigitis, delusions, hemiplegia, etc., are often found. For the same reason we have the pneumonic, the gastralgic, and the hemoglobinuric types. Heroic doses of quinine should be administered by the hypodermic needle or by Bacelli's intravenous method.

Malaria is insidious, but daily examination of the tongues of soldiers will enable one to predict an attack and by prompt administration of quinine to abort it. By this daily examination over fifty per cent. of malarial paroxysms can be aborted, and by heroic administration of the drug fully ninety-five per cent. can be cured. Boil all water used, avoid unnecessary turning over of the soil, have sleeping apartments dry and beds fully two feet from the ground, and, above all things, do not let a soldier do guard duty at night on an empty stomach. A cup of hot coffee or grog should be served.

Dysentery, a disease which killed last year in Cuba twelve thousand soldiers and permanently invalided as many more, deserves a most careful consideration. It usually comes on under the form of a most innocent diarrhea. Suddenly large stools at short intervals supervene, odorless and of a grayish or yellowish color. The first stool may be accompanied by griping, but the reverse is usually the case. In some cases this condition lasts for days or weeks. The patient does not quit his work but contents himself with home remedies. In the great majority of cases, however, the patient presents himself with every evidence of marked debility. He complains of no particular pains, sleeps well, no elevation of temperature, tongue at times clear but usually covered with a thick muddy coat. The most careful examination fails to reveal a grave symptom, yet this disease gives the highest death-rate of all endemic tropical diseases. The first attack is usually recovered from, with good attention, though the recovery is always slow and invariably interrupted by many relapses, but should there be a second or third attack the case is sadly different. He may sink into a chronic and hopeless cachexia or a fever lit up and the end hastened. In the latter case the stools change, becoming frequent and livid in color, and violent cramps occur. The tongue becomes red and pointed, insomnia sets in, there are vomitings and cold sweats, the pulse thready and scarcely perceptible—*c'est fini.*

Many of these cases would recover if taken in time, but soldiers hate the hospital and will often conceal the disease for days. Therefore, treat all cases, especially diarrhea, quickly, actively and continuously.

This disease often takes a gangrenous form. You will recognize it by the nose as well as eye. The terrible tenesmus of the first few hours is fearful to witness, yet some of the cases, strange as it may appear, recover. The liver is always involved and should be closely watched. Chills at intervals, especially at night, intermitting fever, pain in right hypochondrium or right shoulder nearly always denote abscess. An aspirating needle will confirm your diagnosis, which, when once made, leaves you no option but evacuation. A very free opening should be made. If the hepatitis is acute and not suppurative you must endeavor to cause the subsidence of the hyperemia by the administration of small doses of calomel, by dry cups and by a strict milk diet for a few days. A most valuable remedy is the nitro-hydrochloric baths night and morning. Take one ounce of the undiluted acid to one or two gallons of warm water. Place the feet in the bath and sponge the legs, inside of the thighs and the region of the liver for fifteen minutes. If the bath bites too much, diminish the quantity of acid. The treatment of acute amoebic dysentery will vary somewhat according to the case. Of all remedies, ipecac has given the best results and opium the worst. For ipecac to be effective it must be given in the first few days before destruction has occurred. Powdered ipecac rapidly loses its virtue in hot climates, so use only freshly powdered roots. A bolus containing from thirty to sixty grains is the usual dose. Thirty drops of laudanum should be administered half an hour before and a sinapism applied over the epigastrium. The patient should be kept in bed and but little fluid allowed while taking the ipecac. This dose should be repeated in from four to six hours for one or two days. One dose of from ten to fifteen grains of the sulphate of quinine should be given for four or five days in every case of dysentery unless the microscope fails to detect the plasmodium malariae.

When the character of the stools change, subnitrate of bismuth in from fifteen to thirty-grain doses seems to have a happy effect; also salol in five-grain doses. When the cases become subacute good results are obtained from a high enemata of nitrate of silver, thirty grains to the quart, and not less than a quart should he given, preferably two, and should not be repeated oftener than once a day.—*Atlantic Medical Weekly.*

